# Re-emergence of tularemia in Germany: Presence of *Francisella tularensis *in different rodent species in endemic areas

**DOI:** 10.1186/1471-2334-8-157

**Published:** 2008-11-17

**Authors:** Philipp Kaysser, Erik Seibold, Kerstin Mätz-Rensing, Martin Pfeffer, Sandra Essbauer, Wolf D Splettstoesser

**Affiliations:** 1 Bundeswehr Institute of Microbiology, Neuherbergstrasse 11, D-80937 Munich, Germany; 2 German Primate Center, Kellnerweg 4, D-37077 Göttingen, Germany; 3 Institute of Medical Microbiology, Virology and Hygiene, University Hospital Rostock, Schillingallee 70, D-18057 Rostock, Germany

## Abstract

**Background:**

Tularemia re-emerged in Germany starting in 2004 (with 39 human cases from 2004 to 2007) after over 40 years of only sporadic human infections. The reasons for this rise in case numbers are unknown as is the possible reservoir of the etiologic agent *Francisella (F.) tularensis*. No systematic study on the reservoir situation of *F. tularensis *has been published for Germany so far.

**Methods:**

We investigated three areas six to ten months after the initial tularemia outbreaks for the presence of *F. tularensis *among small mammals, ticks/fleas and water. The investigations consisted of animal live-trapping, serologic testing, screening by real-time-PCR and cultivation.

**Results:**

A total of 386 small mammals were trapped. *F. tularensis *was detected in five different rodent species with carrier rates of 2.04, 6.94 and 10.87% per trapping area. None of the ticks or fleas (n = 432) tested positive for *F. tularensis*. We were able to demonstrate *F. tularensis-*specific DNA in one of 28 water samples taken in one of the outbreak areas.

**Conclusion:**

The findings of our study stress the need for long-term surveillance of natural foci in order to get a better understanding of the reasons for the temporal and spatial patterns of tularemia in Germany.

## Background

Tularemia is caused by the gram-negative rod *Francisella (F.) tularensis *which is confined to the Northern Hemisphere, where small mammals such as rabbits, hares and voles are known to be involved in enzootic transmission cycles [1]. *F. tularensis *is regarded as a category A biological agent according to the classification of the CDC (Centers for Disease Control and Prevention, Atlanta, GA, USA).

The last major outbreaks among humans in Germany took place in the late 1950s [2]. Since then a sharp decline in cases of tularemia was seen with less than five per year between 1960 and 2004 [3]. Likewise only four cases of tularemia among hares and rabbits were reported to the German authorities between 1983 and 1992 followed by a period without reports until 2004 [4] when tularemia was the cause of an outbreak among primates in ethological research facilities in Sennickerode (SEN) [3] and in 2005 in Göttingen (GÖ) [5]. Most strikingly, tularemia had never been described in both areas before. In November 2005 finally, the largest outbreak of human tularemia in Germany for 40 years has occurred in a group of hare hunters near the city of Darmstadt (DA) where 11 people got infected including one fatality [6]. Altogether a total of 39 human cases occurred in Germany from 2004 to 2007 [7]. All three areas were investigated in our pilot study in order to detect possible animal reservoirs of *F. tularensis*.

## Methods

**Figure 1:**
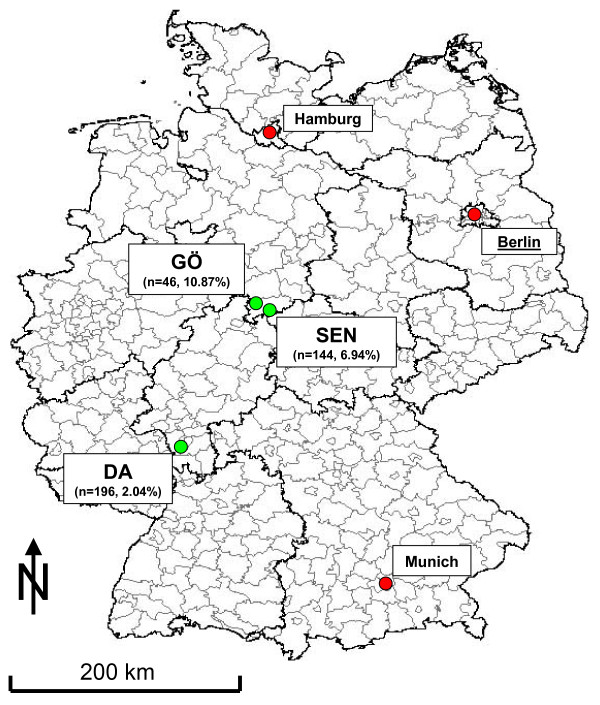
**Study sites, Germany, 2004–2006**. Green dots indicate the investigation areas, red dots the three largest German cities for orientation. The number of small mammals caught and the *F. tularensis *detection rate are shown in parenthesis.

**Figure 2:**
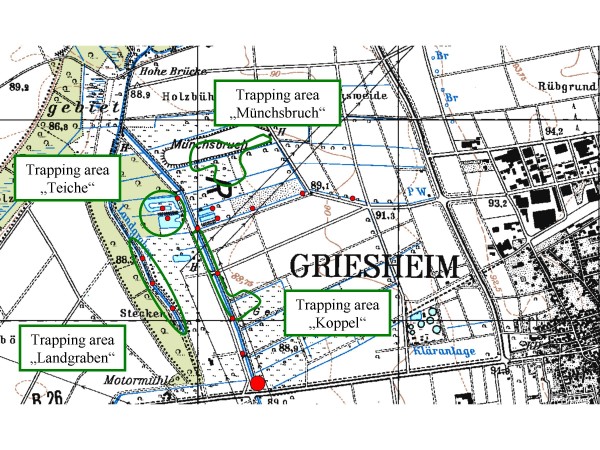
**Distribution of rodent trapping areas (marked in green) and water sample collection sites (red dots) in an exemplary area (DA)**. The large red dot indicates the water sample tested positive for *F. tularensis*.

Live-trapping of small mammals was performed in all three distinct endemic areas (Figure 1) in June 2005 (SEN, area of approximately 50,000 m^2^), in September 2006 (GÖ, approx. 30,000 m^2^) and in October 2006 (DA, approx. 1.2 km^2^) – each within a time period of six to ten months after the initial outbreaks. The endemic areas were divided into up to five trapping areas – depending on size of the area and differences in vegetation (Figure 2).

Small mammals were decoyed and live-trapped with Sherman traps [8], ectoparasites were combed off and – where possible – blood samples were collected by heart-puncture under anaesthesia. After necropsy the species was mainly determined by phenotypic differentiation, in some cases by a PCR targeting the partial mitochondrial cytochrome B gene as described before [8]. Tissue samples of approximately 0.5 g of liver and spleen were homogenized with 0.9 ml of phosphate-buffered saline (PBS) using the FastPrep™-system (QBiogene, Heidelberg, Germany). An aliquot of 20 μl was then lysed with 180 μl of lysis buffer containing proteinase K as previously described [3] and 5 μl were tested in duplicate using a real-time-PCR for the presence of the 16S rRNA-gene of *F. tularensis *(LightMix^®^-Kit *Francisella tularensis*, TIB MOLBIOL, Berlin, Germany). Positive samples were confirmed using a specific *fopA*-real-time-PCR (modified according to [9]). Conventional PCRs targeting a 30 bp-deletion in the Ft-M19 locus [10] and the region of differentiation 1 (RD1, [11]) followed to determine subspecies identity. Cultivation and isolation of the agent was attempted only with liver and spleen homogenates previously tested positive for *F. tularensis *by PCR (10 μl in dilution streak technique on Heart-Cysteine-, Columbia- and Thayer-Martin-agars and 50 μl spread evenly on Heart-Cysteine-agar). Confirmation after growth on agar was done using the above chain of PCR-based methods combined with typical biochemical and morphologic features. Retain samples of all liver and spleen homogenates were stored for possible future use.

Serum samples were examined using an ELISA based on *F. tularensis *LPS as previously described [12]. A highly specific western blot was used as a confirmatory assay [12].

Ticks and fleas (n = 432) were pooled to a maximum of 20 per pool, homogenized and analysed by PCR as described above. Tick species were not determined due to the pilot study character of the investigations. The collection of ticks and fleas was done from the animal itself and by flagging in the small mammal trapping areas defined for every endemic area (Figure 2).

For water samples (collected throughout the entire DA endemic area, Figure 2), 1 ml of each sample was sterile-filtered (1 μm filter) to eliminate suspended matter, centrifuged with 20,000 g for 10 minutes and re-suspended in 50 μl of PBS, 20 μl were then lysed and tested by PCR as described above.

## Results

A total of 386 small mammals were captured during the study, the overall carrier rate with *F. tularensis *as determined by the detection of specific DNA was 4.92% (n = 386, 95% confidence interval: 2.76 – 7.08%, range: 2.04 – 10.87%) in the three endemic areas (Table 1). The infected species were bank voles (*Myodes glareolus; *4.49%), water voles (*Arvicola terrestris; *15.0%), field voles (*Microtus agrestis; *10.0%), common voles (*Microtus arvalis; *8.0%) and yellow-necked fieldmice (*Apodemus flavicollis; *2.9%). We found a significant difference in the infection rate among the three investigation sites DA, GÖ and SEN (Χ^2 ^= 7.34, degrees of freedom = 2, p = 0.025), but not among the five positive rodent species (Χ^2 ^= 6.91, degrees of freedom = 4, p = 0.141).

**Table 1:** Detection of *F. tularensis *in different small mammal species

	**No. Positive/No. Detected (%)**			
	
**Small mammal species**	**Darmstadt (DA)**	**Göttingen (GÖ)**	**Sennickerode (SEN)**	**Total (DA, GÖ, SEN)**
*Apodemus agrarius*	-/-*	0/3	-/-	0/3
*Apodemus flavicollis*	1/30 (3.33)	0/11	1/28 (3.57)	2/69 (2.90)
*Apodemus sylvaticus*	0/52	-/-	-/-	0/52
*Arvicola terrestris*	0/3	-/-	6/37 (16.22)	6/40 (15.00)
*Microtus agrestis*	0/1	1/8 (12.5)	0/1	1/10 (10.00)
*Microtus arvalis*	0/3	1/11 (9.09)	1/11 (9.09)	2/25 (8.00)
*Myodes glareolus*	3/103 (2.91)	3/13 (23.08)	2/62 (3.23)	8/178 (4.49)
*Crocidurina spec*.	0/1	-/-	0/1	0/2
*Sorex spec*.	0/3	-/-	-/-	0/3
Not specified	-/-	-/-	0/4	0/4
Total	4/196 (2.04)	5/46 (10.87)	10/144 (6.94)	19/386 (4.92)

* -/-, no animals captured				

The distribution of *F. tularensis *detection in different small mammal species in the three study sites in absolute and relative numbers is shown. An animal was considered positive for *F. tularensis *when two independent PCR assays demonstrated *F. tularensis*-specific DNA and/or when cultivation of *F. tularensis *was successful.

In none of the 432 ectoparasites from all three study sites *F. tularensis*-specific DNA could be detected.

Strains of *F. tularensis *were isolated from two water voles from the SEN area. Cultural identification, antibiotic resistance typing and PCR results showed that both strains belonged to subspecies *F. tularensis holarctica*, biovar I. None of 186 small mammals (including sera from 15 of the 19 infected mice, no insectivores) from which serum was available showed specific antibodies against *F. tularensis*.

Of the 28 water samples collected in the DA investigation, one sample tested positive for DNA of *F. tularensis *(Figure 2, large red dot). This sample was taken on a spot with floating water of a small river, about 300 m from the next rodent capture site.

The habitats in which the outbreaks occurred consist mainly of alluvial, forest-like field biotopes surrounded by areas of intensive agriculture in Göttingen (GÖ) and Sennickerode (SEN) and also sinuosities of the river Neckar near Darmstadt (DA). Habitat as well as geographic and climatic parameters were very similar to areas in the Czech Republic which seem to favour long-term persistence of *F. tularensis *[3]. In the region of Griesheim (DA investigation, years 1991–2007) the elevation above sea level was 90 m, the mean annual air temperature was 10.9°C and the mean annual precipitation 610 mm (for data on GÖ and SEN see [3]). Except for the mean annual sunshine duration – which is usually lower throughout entire Germany – these characteristics are in accordance with the model suggested in [13].

## Discussion

Human tularemia gained importance in Germany in the years after World War II [2], a total of about 715 cases were notified from 1949–2008 [14]. No systematic investigation on possible reservoirs of tularemia in endemic areas has been published for Germany so far. Public interest in tularemia increased in the last four years due to small localized outbreaks in the areas investigated in this study. Data on the prevalence of *F. tularensis *in rodents as the suspected natural reservoir is of significant importance to German public health authorities in the endemic areas to provide a risk assessment for persons inhabiting or working in these areas.

Our study demonstrates that natural foci do exist in Germany with incidences of *F. tularensis *in rodents of up to 10% in certain foci. The fact that no specific antibodies to *F. tularensis *were found in any of the infected or uninfected animals argues in favour for the traditional view that *F. tularensis *seems to be mainly fatal for infected rodents. However trapping indices in the investigations were good (e. g. in the DA investigation 65 animals/day using 200 traps) suggesting that a rather low prevalence may compensate the high lethality. The overall carrier rate of 4.92% is strikingly similar to the 4.76% found among different rodent species in a recently published study from endemic areas in China [15]. Although the route of transmission to hares or humans still remains unclear, *F. tularensis *seems to have a broad host range within small mammals. Because of the complete lack of seroprevalence found, none of them seem to represent a reservoir host for *F. tularensis*. No tick or flea pool tested positive for the presence of *F. tularensis *either. These findings are supported by the negative testing for *F. tularensis *of more than 2,000 ticks (own unpublished data) over the last years from several areas of Germany, suggesting that neither ticks nor fleas seem to ingest *F. tularensis *and thus are unlikely to be involved in its maintenance and transmission in Germany. These results are in strong contrast to other European countries like Slovakia or the Czech Republic where carrier rates of more than 10% were reported in ticks, mainly *Dermacentor reticulatus*, in some endemic areas [16]. This may be due to the fact that *Ixodes ricinus *is the predominant tick in Germany and reports of *Dermacentor reticulatus *are sparse (only 2126 between September 2004 and May 2006 in a nationwide study [17]). Water on the other hand seems to be a possible route of infection, since we were able to find *F. tularensis *DNA in a water sample for the first time in Germany. This is paralleled by studies from Scandinavia where water was found to be the source of *F. tularensis *infections [18].

## Conclusion

From 2005 to 2008 tularemia in hares (*Lepus europaeus*) was reported from 5 out of 16 German federal states. With 20 human tularemia cases in 2007 the highest number in Germany for almost 50 years was notified [7]. This significant change in the incidence of tularemia within the past four years underlines the need for long-term surveillance of natural foci in order to get a better understanding of the reasons for the temporal and spatial patterns of tularemia in Germany.

## Competing interests

The authors declare that they have no competing interests.

## Authors' contributions

PK planned and carried out the investigation in DA, performed the data analysis of all three areas and drafted the manuscript. ES planned and conducted the investigation in GÖ and took part in laboratory testing/data analysis. KMR assisted in planning and took part in the GÖ investigation. MP assisted in planning and took part in the GÖ investigation and laboratory testing. SE took part in the SEN investigation and laboratory testing. WDS coordinated all three investigations (including laboratory testing), planned and conducted the SEN investigation and took part in the DA investigation. All authors contributed to the study design, the preparation of the manuscript and also read and approved the final manuscript.

## Pre-publication history

The pre-publication history for this paper can be accessed here:


